# Vascular Surgery, Quo Vadis?

**DOI:** 10.3389/fsurg.2014.00030

**Published:** 2014-07-30

**Authors:** Stavros Kakkos

**Affiliations:** ^1^Department of Vascular Surgery, University of Patras Medical School, Patras, Greece

**Keywords:** vascular surgery, aneurysm, varicose veins, peripheral arterial disease, endovascular

Vascular surgery, currently a mono-specialty in a large number of countries, is dedicated to the diagnosis and management of all aspects of vascular disease (arterial, venous, and lymphatic), excluding the heart and brain vessels. Vascular diagnosis is complemented by non-invasive tests that are frequently performed by the managing physician, regardless of the intention for interventional treatment. Non-invasive tests, originally limited mainly to vascular ultrasound but now expanded to include computed tomography and magnetic resonance angiography, have largely reduced the need for arteriography and its attendant risks, preserving its diagnostic ability, and even improving preoperative planning and patient selection.

The dawn of the new millenium found Vascular Surgery strengthened by the FDA approval of endografts for endovascular aneurysm repair (EVAR), a minimally invasive treatment that proved to be a revolution for the treatment of abdominal aortic aneurysms (AAA). It was clear at that point that a decade of research was successfully translated into clinical practice; however, the adoption of EVAR was really supported by trial evidence that became available the following years (1). Stretching the anatomical indications of EVAR and the need for endless follow-up has been a real challenge, partially addressed by new types of endografts. Around the same time, endoluminal vein ablation (ELVA) of the saphenous veins was also approved. Being further refined as new minimally invasive technology is invented, ELVA has replaced a large number of stripping procedures worldwide. Other areas where endovascular techniques have expanded our treatment armamentarium include the peripheral arteries, in order to prevent limb loss, deterioration of quality of life or death. Drug eluting stents and balloons, covered stents and improved atherectomy devices have been introduced into clinical practice.

An area of active debate is the optimum management of extracranial carotid artery stenosis, where endarterectomy is still considered the standard of care, but judicious use of angioplasty and stenting (CAS) might improve outcomes in symptomatic patients. The availability of modern medical treatment has challenged the need for intervention in most patients with asymptomatic carotid artery stenosis and randomized controlled trials (CREST-2) are underway. Selective use of intervention in high-risk groups at a low peri-procedural risk and with sufficient life expectancy is required (2, 3).

Vascular surgeons are actively involved in all kinds of trauma involving the blood vessels and also when these are thrombosed, including deep-vein thrombosis. The advent of new oral anticoagulants is expected to simplify the management of venous thromboembolism (4), but the post-thrombotic syndrome (PTS) is expected to be a burden for affected patients, unless pre-emptive thrombus removal becomes popular (5). Finally, vascular access for hemodialysis has been improved with efforts toward preferential construction of native arteriovenous fistulas and increased longevity of maintenance procedures performed for access complications being fruitful; however, there is an unmet need for further improvements, necessary for this challenging patient population.

The present Specialty Grand Challenge article officially launches the Vascular Surgery Section of Frontiers in Surgery. The goal is to provide a unique open access forum for publication and rapid dissemination of important research covering the entire spectrum of Vascular Surgery.

## Conflict of Interest Statement

The author declares that the research was conducted in the absence of any commercial or financial relationships that could be construed as a potential conflict of interest.
